# Detecting fetal growth restriction with customised assessment: Is the jury still out?

**DOI:** 10.1371/journal.pmed.1004028

**Published:** 2022-06-21

**Authors:** Kate Duhig, Jenny Myers

**Affiliations:** Maternal & Fetal Health Research Centre, Division of Developmental Biology & Medicine, Faculty of Biology, Medicine and Health, University of Manchester, Manchester, United Kingdom

## Abstract

In this Perspective, Kate Duhig and Jenny Myers discuss strategies to improve the detection of abnormal fetal growth trajectories in the antenatal period.

Strategies to improve the detection of abnormal fetal growth trajectories in the antenatal period have received deserved attention, and much effort has focused on the development of fetal growth centile charts. In an accompanying research study in *PLOS Medicine*, Vieira and colleagues assess customised growth charts devised as part of the Growth Assessment Protocol (GAP) against standard care across 13 hospitals in England, in the DESiGN cluster randomised trial [1].

Population-standard centiles charts are underpinned by the theory that fetal growth potential is comparable across populations and that aberrant growth may be attributed to environmental and nutritional factors. The alternative approach of fetal growth “customisation” argues that adjusting for maternal characteristics and parity can improve the precision of fetal growth restriction (FGR) detection, accounting for genetic factors that enthusiasts believe may significantly influence fetal growth [2]. The GAP [3] was developed by the Perinatal Institute for the detection of small for gestational age (SGA) fetuses with customisation and includes a programme of risk assessment, training, and accreditation of the use of customised growth charts in antenatal care settings. The GAP programme customises fetal growth trajectories by adjusting for maternal height, weight, parity, and ethnicity. To date, there has been a lack of studies that prospectively compare the performance of population-based and customisation-based fetal growth assessment for either the detection of SGA or various other downstream adverse perinatal outcomes.

The DESiGN trial had the aim of determining whether implementing GAP would improve antenatal detection of SGA. Thirteen hospital clusters in England were randomised to implementation of the GAP protocol or to standard care. Nearly 25,000 women were included in the analysis. The authors found no difference in the detection of SGA with GAP compared to standard practice. An observed reduction seen in the stillbirth rate in women exposed to the GAP intervention was 1 of 26 tested secondary outcomes with no statistical correction for multiplicity. Given that the detection of SGA was not improved with GAP (the intended mechanism of adverse outcome reduction), it seems possible that this is a type I error rather than a true effect.

The pragmatism of the trial methodology made it a feasible endeavour, but standard care was hard to define, impossible to protocolise, and difficult data to capture. Importantly, scan frequency increased in both arms of the trial, and it was clearly unintentional that the largest increase in scan frequency was seen with standard care. In addition, there was poor fidelity with adherence to the GAP protocol: Only 8.5% of women with risk factors for SGA had at least the minimum number of scans recommended by GAP. External factors beyond the control of the trial team included the national rollout of the “Saving Babies Lives (SBL) Care Bundle” implemented by NHS England [4]. Explicit aims of SBL were to improve the detection of FGR and to reduce perinatal mortality. While the standard care clusters in the DESiGN trial were exempt from compliance with this aspect of the care bundle for the trial duration, units were exposed to other aspects of the SBL intervention that included raising awareness of fetal movements, effective fetal monitoring in labour, and smoking cessation strategies. It seems likely that clinical practice may have been affected by the SBL recommendations on FGR surveillance and that the standard care arm would have been influenced by this shift in national public health policy. It cannot be known whether the outcome of this study might have been different without the introduction of SBL and with better adherence to the GAP protocol. It is possible that the intervention may have improved FGR detection in an efficacy trial, free from shifts in care trends and policy changes, and with a more rigidly prescriptive protocolisation of “standard care.”

FGR is thought to be the most significant single risk factor for stillbirth, particularly when it remains undetected [5,6]. Stillbirth rates in the United Kingdom are falling, which supporters of GAP have attributed, at least in part, to the introduction and increasing uptake of GAP across England [7]. Critics have remonstrated that the stillbirth rate is mirrored in Scotland, where GAP implementation over the same timescale was much more limited and believe that the temporal decline in stillbirth rates are merely coincidental [8]. Other initiatives concurrently implemented that may have contributed to the observed decline in stillbirth include strategies to increase awareness surrounding fetal movements and legislation that made smoking illegal in all public places in England and has driven smoking rates down nationally. In 2017, the British Government announced a maternity strategy to halve the risk of stillbirth by 2025 [9]. The World Health Organization regard reducing stillbirth globally a major target, highlighted via the Every Newborn Action Plan [10]. Undoubtedly, our specialty needs to improve detection of SGA, and to target interventions to reduce the risk of stillbirth, often referred to as the “silent epidemic” in obstetrics.

A variety of risk factors for stillbirth can be determined at pregnancy confirmation, but individual factors attribute such a small amount to overall risk that they have a limited predictive value [11]. Nulliparous women are at increased risk of stillbirth, but prediction in this group is particularly tricky, given that the strongest predictors are poor outcomes in previous pregnancies. Managing SGA is clinically challenging; there is no treatment, and the only strategy is surveillance and attempts to optimally time delivery. It is important that strategies to improve detection of SGA should include protocolisation of the subsequent management of these complicated pregnancies. Babies born to women of Black ethnicity are significantly more likely to experience adverse outcomes and have higher rates of stillbirth across all gestations [12]. Adverse perinatal outcomes such as preterm birth and SGA are higher in Black and Asian women and those with underlying vascular disease [13]. Globally, stillbirth disproportionally affects women from minority ethnic communities, women who live in poverty, women with complex physical and mental health needs, and women exposed to domestic and intimate partner violence [5,14,15]. Systemic biases and factors associated with stillbirth may prevent women with complex physical and social needs receiving adequate care and mirror the health inequalities experienced by women who die during pregnancy [16].

It is without doubt that we must keep stillbirth reduction as a public health priority globally, work urgently to reduce unrecognised FGR, and do more to tackle systemic biases faced by women to prevent babies dying.

## Abbreviations

FGR: fetal growth restriction
GAP: Growth Assessment Protocol
SBL: Saving Babies Lives
SGA: small for gestational age
